# Comment on Balwierz et al. Potential Carcinogens in Makeup Cosmetics. *Int. J. Environ. Res. Public Health* 2023, *20*, 4780

**DOI:** 10.3390/ijerph21020160

**Published:** 2024-01-31

**Authors:** Jacques-Aurélien Sergent, Juergen Nolde, Klaus Weber, Tobias B. Schuster, Valerie Moise, Wolfgang Keller, Jenny Franklin

**Affiliations:** 1 Solvay TERA Unit, 320 Rue de Ransbeek, 1120 Neder Over Heembeek, Belgium; 2 Grace GmbH, In der Hollerhecke 1, 67547 Worms, Germany; juergen.nolde@grace.com; 3 K. Weber Consulting GmbH, Buchsweg 4, CH 4625 Oberbuchsiten, Switzerland; kweber@anapath.ch; 4 AnaPath Services GmbH, 4410 Liestal, Switzerland; 5 Smart Materials, Evonik Operations GmbH, 63457 Hanau, Germany; tobias.schuster@evonik.com; 6 Cabot Performance Materials Belgium, 78 Rue Prevochamps, 4860 Pepinster, Belgium; valerie.moise@cabotcorp.com; 7 Regulatory Affairs, Wacker Chemie AG, 84489 Burghausen, Germany; wolfgang.keller@wacker.com; 8 PQ Silicas UK Ltd., 4 Liverpool Road, Warrington WA5 1AB, UK

The article by Balwierz et al. [1], entitled “Potential carcinogens in Makeup Cosmetics”, provides a review of the presence of some selected cosmetics ingredients that they identify as potential carcinogens, based on carcinogen classifications developed by the International Agency for Research on Cancer (IARC) [2].

However, we noticed several inaccuracies and misrepresentations related to the substance named “silica” in the publication that we would like to clarify in our comments.

There is a misunderstanding of the identity of “silica” used in cosmetics, which is a form of synthetic amorphous silica, distinct from crystalline silica. Indeed, in Section 4.2.2, Balwierz and his co-authors introduced “Silica (silicon dioxide)” as a compound commonly found on earth. 

The author refers to the CoSing Database, the European Commission database for information on cosmetic substances and ingredients. However, the author failed to identify the following elements.

SILICA is the INCI—International Nomenclature for Cosmetic Ingredient—used under the European Cosmetic Products Regulation (CPR) to characterize a specific form of Synthetic Amorphous Silica (SAS) being produced via a thermal route, also called pyrogenic SAS [3]. Through the entire paragraph dedicated to silica, there is an apparent confusion between the chemical name silica/silicon dioxide and the INCI name, “SILICA”. The wording “SILICA” under the European Cosmetics Products Regulation should be understood/read as the pyrogenic SAS.

In addition, the author allocates potential carcinogenicity to “silica” as a substance, which is not accurate.

Silicon dioxide/silica with CAS number 7631-86-9 describes all forms of SiO_2_ (See Figure 1). These forms could be either crystalline or amorphous. Among the crystalline forms, we can identify quartz (CAS 14808-60-7), cristobalite (CAS 14464-46-1) or tridymite (CAS 15468-32-3). The International Agency for Research on Cancer (IARC), a member of the World Health Organization (WHO), classified silica as Group 1 (carcinogenic to humans) for crystalline forms, while it is Group 3 (not classifiable as to its carcinogenicity to humans) for the amorphous form [2]. [It is noted that in the Abstract, the author incorrectly refers to the Insecticide Resistance Action Committee (IRAC) instead of the International Agency for Research on Cancer (IARC).]

Under the family of the amorphous forms, we can identify naturally occurring forms, like diatomite (CAS 61990-53-2)—known as SOLUM DIATOMEAE under CosIng—calcined (CAS 91053-39-3), or flux calcined (CAS 688855-54-9); incidental ones, like fused silica (CAS 60676-86-0) or silica fume (69012-64-2); and those which are synthetically produced, mainly via two different manufacturing routes: the thermal one (pyrogenic SAS 112945-52-5) and the wet route (precipitated SAS and silica gel CAS 112926-008) [4].

Synthetic amorphous silica does not share the same toxicological profile as crystalline silica (as noted above, it is not classified as carcinogenic by IARC). There are no makeup cosmetics containing crystalline silica, as is incorrect ly stated by Balwierz and co-authors. The entire section about the crystalline form of SiO_2_ is therefore not relevant to the cosmetics market. (Is Silica Powder Safe In Mineral Makeup Products? Available online: https://www.sterlingminerals.com/is-silica-powder-safe-in-mineral-makeup-products/, accessed on 2 October 2023).

To note also, the authors cited multiple references to silicon dioxide nanoparticles. However, it should be noted that the wet route and pyrogenic SAS are nanostructured materials with constituent particles at the nanoscale fused together in aggregates typically larger than 100 nm; the substance is therefore available as an agglomerated aggregated material with a dimension up to the micron or millimeter scale. The release of “constituent” particles from aggregates is thermodynamically very difficult, even if fragments of materials can be obtained if very high energy is applied for a sufficient amount of time [5].

In Table 2 [1], the authors overall allocate a risk of carcinogenicity and pulmonary fibrosis to all makeup products containing “silica”. A basic toxicology principle is to consider the route of exposure before associating a hazard to a use. Should a real ‘inhalation exposure risk’ be identified, this would not be relevant for cosmetics applied dermally or orally. Inhalation exposure is negligible for makeup or skincare products [6], unless they are sprayed. Thus, even if crystalline silica was used in cosmetics, it would not, based on the conclusions of the IARC, create a risk in oral or dermal applications as the carcinogenic potential is only defined through the inhalation route.

Some of the literature cited by the authors is mis-quoted or is not relevant to synthetic amorphous silica. The National Institute of Environmental Health and Safety National Toxicology Program. Silica, Crystalline (Respirable Size)—Report on Carcinogens, 14th ed [7] cited by the authors has been meanwhile replaced by RoC Profile (Silica, Crystalline (Respirable Size), Available online: https://ntp.niehs.nih.gov/ntp/roc/content/profiles//silica.pdf, accessed on 12 December 2023, which also concludes—*Respirable crystalline silica, primarily quartz dusts occurring in industrial and occupational settings, is known to be a human carcinogen based on sufficient evidence of carcinogenicity from studies in humans. Respirable crystalline silica was first listed in the Sixth Annual Report on Carcinogens in 1991 as reasonably anticipated to be a human carcinogen based on sufficient evidence of carcinogenicity from studies in experimental animals; the listing was revised to known to be a human carcinogen in the Ninth Report on Carcinogens in 2000*. But again, this classification is relevant only for the inhalation exposure route; there is no hazard classification of (synthetic) amorphous silica as of today.

Balwierz et al. referred to Ryu et al.’s [8] publication summarizing a 90-day nanoparticle toxicity study after topical exposure according to OECD TG 411 with colloidal silica, a subform of synthetic amorphous silica not used in cosmetic products, with nanoparticles 20 nm in size, but which made a contradictory conclusion. Ryu et al. claimed that *SiO_2_ NPs administered through the dermal route for 90 days by topical application on the skin of the back was safe, without any internal organ damage, up to a dose of 2000 mg/kg in rats* [8], while Balwierz et al. state that *It has been theorized that the reduction in silica particle size would allow them to be more easily accessible to the body through various penetration routes.*

A Danish authority report also concluded that SAS does not penetrate the skin [9].

Hirai et al. [10], cited by the authors, is only a short communication, and the results are rather questionable as the analytical methods used do not allow the firm identification of ‘silica’ particles by TEM. Furthermore, a stability test of the fluorescent marker on the silica surface is not provided, meaning that due to the harsh dispersion method, this study is questionable. A penetration test through mice ears is not a proven skin penetration test either [11].

Given the many caveats associated with the publication and more precisely the non-scientifically justified silica-related hazard, and the fact that SAS (SILICA and HYDRATED SILICA (INCI of precipitated SAS)) has been used for many years and found to be safe by several authorities (EU, US FDA, CFDA, etc.), this paper requires detailed corrections or should be withdrawn as the IARC systematic is wrongly used and the conclusions made about the products are unjustified.

## Figures and Tables

**Figure 1:**
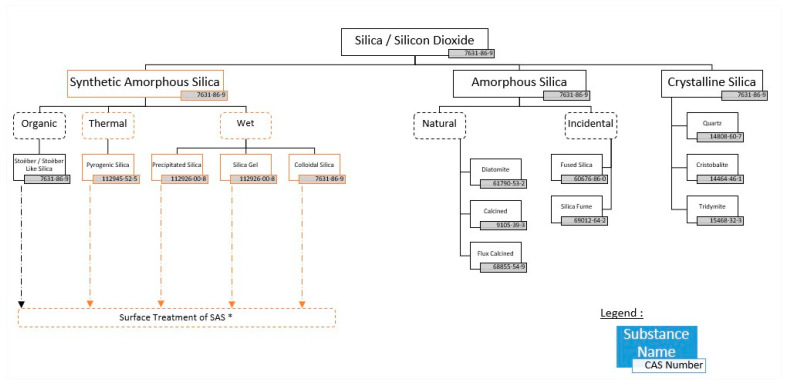
Silica polymorphs [ASASP based on ECETOC JACC 51]. * SAS stands Synthetic Amorphous Silica.
